# An 81-Year-Old Geriatric Patient with Metastatic Pancreatic Cancer Demonstrating Excellent Response and Well Tolerance to NALIRIFOX: A Case Report and Literature Review

**DOI:** 10.3390/reports8020069

**Published:** 2025-05-15

**Authors:** Bayan Khasawneh, Abdullah Esmail, Ebtesam Al-Najjar, Seif El Beheary, Maen Abdelrahim

**Affiliations:** 1 Section of GI Oncology, Houston Methodist Neal Cancer Center, Houston Methodist Hospital, Houston, TX 77094, USAaesmail@houstonmethodist.org (A.E.);; 2 College of Natural Sciences, University of Houston, Houston, TX 77204, USA

**Keywords:** pancreatic cancer, NALIRIFOX, PDAC, toxicity and tolerability

## Abstract

**Background and Clinical Significance:** Pancreatic cancer was the third leading cause of cancer-related mortality in the United States in 2020 after lung and colorectal cancers. The prevalence of pancreatic cancer has been increasing and is projected to continue rising through 2040, with an estimated 355,317 additional cases expected. We present the case of an 81-year-old patient with metastatic pancreatic ductal adenocarcinoma (PDAC) who tolerated NALIRIFOX for a year with grade 1 adverse events. **Case presentation:** An 81-year-old Asian male presented with abdominal pain associated with weight loss and fatigue. An abdominal computed tomography (CT) scan showed a mass in the body of the pancreas measuring 3.5 cm with an infiltrative appearance invading the retroperitoneum and encasing the splenic artery. A biopsy confirmed poorly differentiated PDAC. The patient received 13 cycles of NALIRIFOX in a palliative setting over the course of one year, demonstrating excellent tolerance aside from minor toxicities, including worsening of pre-existing macrocytic anemia, treatment-related grade 1 neuropathy, diarrhea, and thrombocytopenia. A subsequent CT scan revealed disease progression, and the patient was switched to second-line therapy. However, per his preference, the patient was referred to hospice care and passed away a few days later. **Conclusions:** This case highlights the excellent tolerability of NALIRIFOX in an elderly patient, with minimal adverse events observed, which is uncommon among similar patient populations.

## 1. Introduction

A clear understanding of the burden of cancer is essential for shaping health policy and guiding research priorities. Rates of cancer incidence and prevalence help quantify both economic and societal impacts. In the United States, pancreatic cancer is ranked as the third leading cause of cancer-related deaths in 2020 [1,2]. By 2022, it had caused approximately 467,000 deaths worldwide, accounting for about 4.8% of all cancer fatalities [3,4,5]. Alarmingly, global incidence continues to rise and is projected to increase further, with an estimated 355,000 new cases anticipated by 2040 [6,7].

The pancreas contains both endocrine and exocrine tissue. Its endocrine cells regulate blood glucose through insulin and glucagon, while the exocrine portion releases digestive enzymes. Most pancreatic tumors arise from the exocrine component, particularly in the form of pancreatic ductal adenocarcinoma (PDAC), which is the most common histologic type [8,9,10,11].

Treatment for pancreatic cancer typically involves a combination of surgery, chemotherapy, radiation, and supportive measures. However, when patients are diagnosed at an advanced stage, often with distant metastasis, curative options are limited. In such cases, systemic chemotherapy remains the primary treatment strategy [12,13].

Outcomes remain poor overall. Untreated PDAC patients may survive only a few months, while those receiving aggressive therapy achieve a median overall survival (OS) of roughly 11 months [14,15]. For patients with metastatic disease, the five-year survival rate is estimated at just 3% [16,17]. Several factors contribute to this poor prognosis: late diagnosis, a dense tumor microenvironment, and resistance to traditional chemotherapies [18,19]. Additionally, PDAC tends to spread early due to its anatomical proximity to major vascular structures, which often renders tumors unresectable [20].

Despite years of research, progress has been slow. While a few recent therapies have shown promise, the standard of care has remained largely unchanged. Most approaches still rely on established chemotherapy agents, with only minor adjustments in dose or formulation [21]. Also, patients with similar disease profiles often respond differently to the same treatment, this highlights how much remains unknown about the underlying biology of pancreatic cancer.

FOLFIRINOX, a combination of leucovorin, 5-fluorouracil, irinotecan, and oxaliplatin, has been the standard first-line regimen, known to prolong survival. However, its toxicity is significant, making it unsuitable for many, especially patients who are over a certain age, typically around 80 years. The survival data from the NAPOLI-3 trial [22], which utilized an altered drug regimen, supports this case study. It revealed a sustained response to NALIRIFOX (liposomal irinotecan, oxaliplatin, leucovorin, and 5-fluorouracil) treatment in metastatic PDAC patients. It also showed a more favorable safety profile and improved survival. Even so, severe (grade ≥ 3) adverse events were reported in 71% of patients treated with NALIRIFOX, with a median age of 64 (range 20–85 years; IQR 57–70). Another commonly used regimen, gemcitabine combined with nab-paclitaxel (GEM-NABP), has shown efficacy in metastatic PDAC with a more favorable tolerability profile, particularly in older patients with comorbidities [23].

In this case, report, we describe an 81-year-old cachectic patient with metastatic PDAC who, despite his age and frailty, tolerated NALIRIFOX remarkably well for over a year, experiencing only grade 1 side effects.

## 2. Case Presentation

An 81-year-old Asian male presented with abdominal pain associated with weight loss and fatigue for 2 months. The patient used to be active and exercised on a regular basis but recently became intolerant to exercise. On examination, the patient was cachectic and had a body mass index (BMI) of 17.7 kg/m^2^. However, laboratory results revealed elevated levels of carbohydrate antigen (CA) 19-9 tumor marker of 16,647 U/mL (reference level 0–35 U/mL) and carcinoembryonic antigen (CEA) was 13.8 ng/mL (reference level 0–3.8 ng/mL). The patient used to be a smoker but quit 30 years ago. The family history was notable for several cases of cholangiocarcinoma.

**Figure 1:**
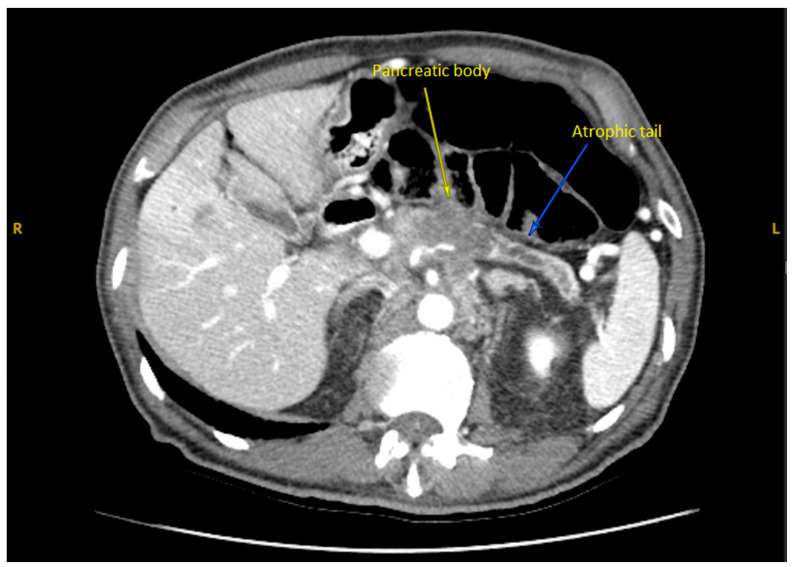
Axial CT Abdomen Pelvis with contrast showed the body of a pancreas tumor.

**Figure 2:**
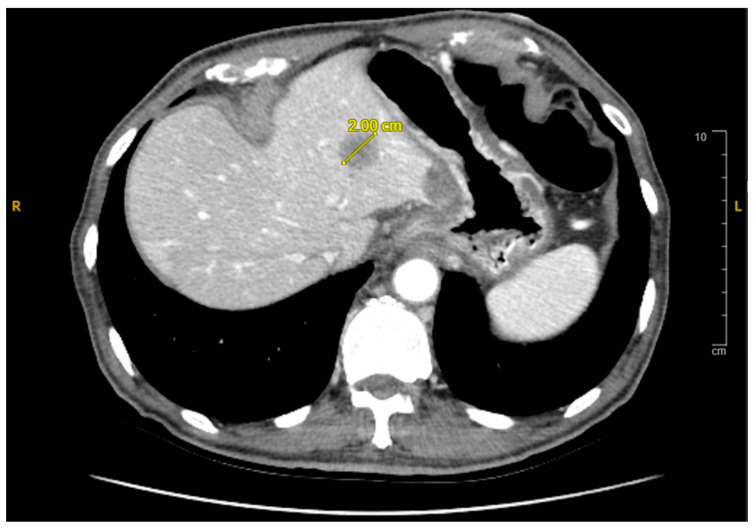
Axial CT Abdomen Pelvis with contrast showing a 2.0 cm lesion in the liver consistent with metastasis.

A subsequent abdominal computed tomography (CT) scan revealed a mass in the body of the pancreas measuring 3.5 cm with an infiltrative appearance invading the retroperitoneum and encasing the splenic artery. It also showed atrophic tail of the pancreas and dilatation of the distal pancreatic duct (Figure 1). A few liver lesions were found to be metabolically active on PET/CT, with the largest measuring 2.0 cm (Figure 2; PET/CT image available upon request). In addition, multiple nodules were noted in the lungs with the largest nodule measuring about 2.0 cm. These findings were suggestive of metastatic disease.

The patient underwent an endoscopic ultrasound-guided fine-needle biopsy of the pancreatic mass, which confirmed poorly differentiated metastatic PDAC. Multiple hypoechoic lesions were identified in the right and left lobes of the liver, with the largest measuring 0.5 × 0.2 × 0.1 cm, confirmed as metastatic PDAC via endoscopic ultrasound-guided biopsy. Molecular tests for KRAS, NRAS, and BRAF, routinely performed at our institution for PDAC, were negative. UGT1A1 testing, which can predict irinotecan-related toxicities, was not conducted as it was not part of the standard protocol at the time [24]. Given the patient’s Eastern Cooperative Oncology Group (ECOG) performance status of 1 and no significant comorbidities other than hypertension and hyperlipidemia. Based on diagnostic findings, the patient was diagnosed with stage IV metastatic PDAC with a primary tumor in the pancreas and metastases to the liver and lungs. The patient’s cognitive function was intact based on clinical evaluation, and he relied on protein supplements to address nutritional deficits. Family members provided strong social support and were actively involved in treatment decision making.

The patient was screened for clinical trials, including NAPOLI-3 or Astella clinics trials, FOLFIRINOX or Nab-paclitaxel were offered as treatment options. After discussing the available treatment options with the patient and his family and based on the clinical judgment of the treating physician, it was decided to proceed with the NALIRIFOX regimen, in alignment with the NAPOLI-3 protocol, and per the patient and family’s preference. The patient subsequently received cycle 1 of NALIRIFOX which included (liposomal irinotecan 50 mg/m^2^ on day 1, oxaliplatin 60 mg/m^2^ on day 1, leucovorin 400 mg/m^2^ on day 1 and 5-fluorouracil 2400 mg/m^2^ continuous infusion over 46 h every 15 days) as palliative setting. At the time of the treatment initiation, the CA 19-9 level was 16,942 U/mL. The patient had excellent tolerance during the first three cycles of therapy. Also, he was encouraged to increase his protein intake by using protein supplements and shakes in addition to dronabinol to help with his low appetite. Routine CT scan assessments were performed every two to three cycles of treatment. A restaging CT scan following three cycles of NALIRIFOX showed an interval reduction in the pancreatic mass with decreased size of the pulmonary and liver metastatic lesions as compared to prior CT scans. CA 19-9 levels gradually decreased to 6946 at cycle 3. A CT scan performed at cycle 5 showed a partial response (PR), with a decrease in the size of pulmonary and hepatic metastases and decreased CA 19-9 level to 634 U/mL. The patient tolerated the regimen well until cycle 5, when grade 1 diarrhea, as reported using the Common Terminology for Adverse Events (CTCAE) version 5.0, was reported and managed with loperamide and diphenoxylate/atropine. Subsequent scans in the following months continued to demonstrate stable disease.

The patient developed grade 1 thrombocytopenia at cycle 8, prompting a precautionary delay in treatment until improvement was observed, as per clinical judgment, before proceeding with the next cycle. Additionally, grade 1 neuropathy, which was managed initially with a dose adjustment of liposomal irinotecan to 60 mg/m^2^, oxaliplatin to 73 mg/m^2^, and 5-FU to 1920 mg/m^2^ (DL-1 per NAPOLI-3 protocol) on cycle 9, day 15 (C9D15). Another dose adjustment of oxaliplatin to 39 mg/m^2^ was made on cycle 10, day 15 (C10D15) per protocol. Gabapentin was prescribed for grade 1 neuropathy management. The patient also had worsening pre-existing macrocytic anemia.

**Figure 3:**
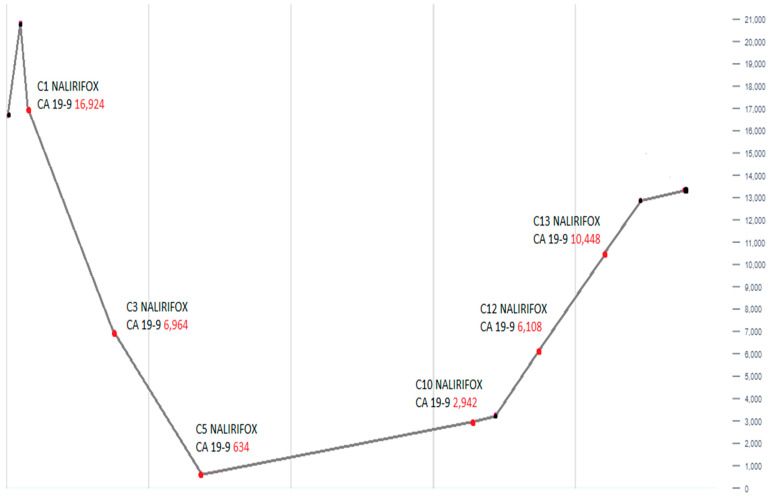
This line graph shows the alterations in serum CA 19-9 levels over the course of treatment with NALIRIFOX. An initial marked decline in CA 19-9 is followed by a gradual and then steep rise, reaching the peak at cycle 13 (C13). This trend demonstrates initial therapeutic response followed by disease progression.

**Figure 4:**
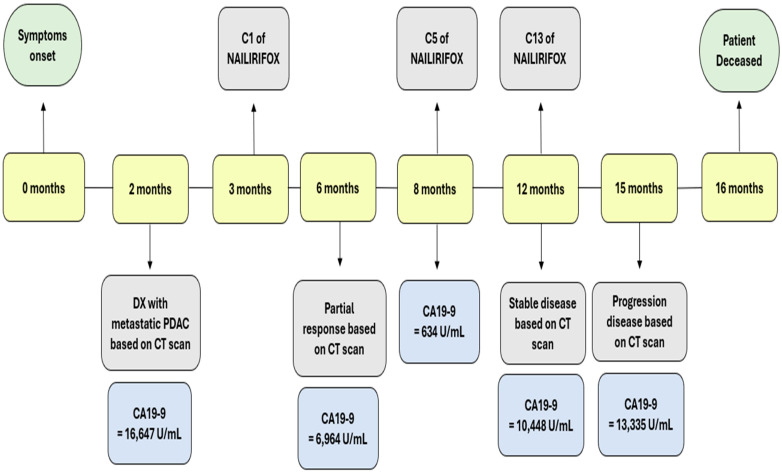
This timeline shows the clinical course of our patient from symptom onset to death over a 16-month period. Key events are marked, including diagnosis (month 2), initiation of NALIRIFOX therapy (month 3), and disease evaluations based on CT scans. Corresponding CA 19-9 levels are shown at each major stage, highlighting an initial biochemical and radiographic response (CA 19-9 decline to 634 U/mL at month 8) followed by a gradual rise in tumor marker levels, consistent with disease progression (CA 19-9 at 13,335 U/mL at month 15). The patient died at month 16.

Follow-up CT scans in the following months revealed a mostly stable disease. However, the CA 19-9 levels began to gradually increase as shown in Figure 3. A follow-up CT scan after cycle 13 indicated the progression of the disease (as per RECIST version 1.1). Consequently, the patient was switched to second-line therapy with GEM-NABP after receiving NALIRIFOX for one year. However, treatment was deferred due to the worsening of the patient’s clinical status. Following treatment discontinuation, the patient’s performance rapidly declined after he developed a stroke, despite being on anticoagulants. Following his preference, the patient was referred to hospice care and passed away within the same week. The timeline in Figure 4 shows a detailed description of the patient’s diagnosis and treatment history.

## 3. Discussion

Pancreatic cancer is primarily diagnosed in older patient populations, with nearly 90% of cases diagnosed after the age of 55 years, and the median age at the time of diagnosis is 70 years. The standard treatment approach for metastatic PDAC includes FOLFIRINOX and gemcitabine with Nab-paclitaxel (GEM-NABP) with the latter being preferred among older patient populations with associated comorbidities due to the overall better tolerability and good efficacy, providing a middle ground for elderly patients.

The NAPOLI-3 trial has demonstrated statistically significant improvements in the OS and the progression-free survival (PFS) in patients with metastatic PDAC who have not received treatment in the metastatic setting. NALIRIFOX was approved by the FDA as a first-line therapy for patients with metastatic PDAC on 13 February 2024. NALIRIFOX use in elderly patients is limited due to a higher risk of adverse events, the associated comorbidities, poor performance status, or cognitive impairment. However, with careful monitoring, NALIRIFOX can still be appropriate for some patients. The NAPOLI-3 trial, which included patients with a median age of 64 years, revealed grade 3–4 adverse events among 87% of patients receiving NALIRIFOX, with 71% considered to be treatment-related, leading to treatment discontinuation in 25% of patients due to toxicity [22].

On the other hand, our 81-year-old patient with metastatic PDAC received NALIRIFOX for one year and demonstrated excellent tolerance for NALIRIFOX with only minimal adverse events. This included grade 1 neuropathy, diarrhea, and thrombocytopenia, as reported using the CTCAE version 5.0, along with worsening of pre-existing macrocytic anemia. Despite the documented side adverse event profile of NALIRIFOX among this patient population in NAPOLI-3 trial, our patient demonstrated remarkable tolerance to this treatment and received the treatment for a period that is well above the median treatment durations which correlated with 24.3 weeks (around 6 months) and a median of 5 treatment cycles. This showcases a novel outlook on the potential tolerability of NALIRIFOX in selected patients and emphasizes the importance of individualized and patient-tailored treatment strategies in the treatment of PDAC.

The absence of KRAS, NRAS, and BRAF mutations, present in over 90% of PDAC cases, is highly atypical and may have contributed to the patient’s exceptional response to NALIRIFOX. Non-KRAS-mutated PDAC may exhibit distinct biological behaviors, potentially influencing chemotherapy sensitivity [25]. However, this unique profile limits the generalizability of our findings to typical PDAC cases.

As an Asian male, the patient’s HLA profile may have influenced drug metabolism or immune response to NALIRIFOX, though specific data linking HLA to PDAC treatment outcomes are limited. In elderly patients, NALIRIFOX tolerability may be enhanced by careful patient selection, considering factors like performance status and comorbidities, compared to younger patients with potentially higher resilience but similar toxicity risks [26].

While CA 19-9 trends correlated with radiographic response and progression, confounders such as biliary obstruction or non-cancer-related inflammation can influence levels. No such conditions were clinically evident in this patient, supporting the reliability of CA 19-9 as a biomarker in this case [27]. However, the patient’s 16-month survival, exceeding the median for metastatic PDAC, likely reflects NALIRIFOX efficacy combined with favorable patient factors, including an ECOG performance status of 1 and minimal comorbidities. The rapid decline in post-treatment discontinuation was driven by a stroke and disease progression, unrelated to prior treatment toxicities.

Ref. [28] is a systematic review and meta-analysis published by Nicchetti et al. in 2024, highlighting that NALIRIFOX and FOLFIRINOX are potentially equally effective as first-line treatments for PDAC, but they are different in their toxicity profiles [28]. Nicchetti et al. also found that both regimens have greater overall response rates and better OS compared to GEM-NABP, with no significant difference in OS between FOLFIRINOX and NALIRIFOX [23,28]. Regarding toxicity, NALIRIFOX was linked to a significantly lower incidence of thrombocytopenia (1.6%) compared to FOFIRINOX (11.8%) and GEM-NABP (10.8%), as well as lower rates of anemia and neutropenia compared to GEM-NABP [28]. However, NALIRIFOX had a slightly higher incidence of diarrhea (20.3%) compared to GEM-NABP (15.7%), though not significantly different from FOLFIRINOX (16.8%). On the other hand, FOLFIRINOX had the highest risk of febrile neutropenia and vomiting, while GEM-NABP was associated with the highest rates of anemia and peripheral neuropathy among the three regimens. These findings align with our case, in which an 81-year-old had exceptional tolerance to NALIRIFOX with minimal toxicities including grade 1 neuropathy, diarrhea, and thrombocytopenia. However, this meta-analysis by Nicchetti et al. comparing NALIRIFOX, FOLFIRINOX, and GEM-NABP may include heterogeneous studies with varying patient populations and protocols, potentially introducing biases. Hence, our case findings, specific to a single elderly patient, should be interpreted cautiously in the context of broader trial data.

Research shows that elderly cancer patients are often under-represented in clinical trials for new cancer treatments. Thus, there is a lack of sufficient studies that provide data on the tolerability and effectiveness of chemotherapy in this age group [24]. In clinical practice, older patients are typically less likely to receive standard chemotherapy regimens compared to younger patients. Moreover, their median OS rates are 6–8 weeks shorter than what is reported in clinical trials [29].

Due to NALIRIFOX acceptable toxicity profile and its high objective response rate, it is particularly perfect for lowering the risk of disease progression while minimizing the toxic effects [28]. Our findings align with this study which utilized NALIRIFOX for an elderly patient. The treatment was well-tolerated, with manageable adverse effects such as grade 1 neuropathy, diarrhea, and thrombocytopenia.

## 4. Conclusions

Our geriatric case demonstrated remarkable tolerance to NALIRIFOX with minimal toxicities, achieving partial tumor response and stable disease before progression. These findings showcase the potential feasibility of NALIRIFOX as a compelling treatment option for selected elderly patients with metastatic PDAC, emphasizing the need for further research to support its feasibility and tolerance in the geriatric population, despite the current concerns regarding its high toxicity profile.

## Data Availability

The data of this case report that supports our results are available upon request from the corresponding author, Maen Abdelrahim.
